# Course of avoidant/restrictive food intake disorder: Emergence of overvaluation of shape/weight

**DOI:** 10.1186/s40337-024-01001-3

**Published:** 2024-05-03

**Authors:** P. Evelyna Kambanis, Christopher J. Mancuso, Kendra R. Becker, Kamryn T. Eddy, Jennifer J. Thomas, Kyle P. De Young

**Affiliations:** 1 Eating Disorders Clinical and Research Program, Massachusetts General Hospital, Suite 200, Boston, MA 02114 USA; 2 Department of Psychiatry, Harvard Medical School, Boston, MA USA; 3 Department of Psychology, University of Wyoming, Laramie, WY USA

**Keywords:** Avoidant/restrictive food intake disorder, ARFID, Anorexia nervosa, Bulimia nervosa, Binge-eating disorder, Diagnostic crossover, Overvaluation of shape/weight, Diagnosis, Assessment, Retrospective study, Longitudinal study

## Abstract

**Background:**

Avoidant/restrictive food intake disorder (ARFID) is a feeding/eating disorder characterized by avoidance/restriction of food intake by volume and/or variety. The emergence of shape/weight-related eating disorder symptoms in the longitudinal course of ARFID is an important clinical phenomenon that is neither robustly documented nor well understood. We aimed to characterize the emergence of eating disorder symptoms among adults with an initial diagnosis of ARFID who ultimately developed other eating disorders.

**Method:**

Thirty-five participants (94% female; *M*_age_ = 23.17 ± 5.84 years) with a history of ARFID and a later, separate eating disorder completed clinical interviews (i.e., Structured Clinical Interview for *DSM-5* – Research Version and Longitudinal Interval Follow-Up Evaluation) assessing the period between ARFID and the later eating disorder. Participants used calendars to aid in recall of symptoms over time. Descriptive statistics characterized the presence, order of, and time to each symptom. Paired samples *t-*tests compared weeks to emergence between symptoms.

**Results:**

Most participants (71%) developed restricting eating disorders; the remainder (29%) developed binge-spectrum eating disorders. Cognitive symptoms (e.g., shape/weight concerns) tended to onset initially and were followed by behavioral symptoms. Shape/weight-related food avoidance presented first, objective binge eating, fasting, and excessive exercise occurred next, followed by subjective binge eating and purging.

**Conclusions:**

Diagnostic crossover from ARFID to another (typically restricting) eating disorder following the development of shape/weight concerns may represent the natural progression of a singular clinical phenomenon. Findings identify potential pathways from ARFID to the development of another eating disorder, highlighting possible clinical targets for preventing this outcome.

## Abstract (plain language summary)

Avoidant/restrictive food intake disorder (ARFID) is a feeding/eating disorder characterized by avoidance/restriction of food intake by volume and/or variety. In contrast to other eating disorders (e.g., anorexia nervosa, bulimia nervosa), dietary restriction in ARFID is not motivated by shape and weight concerns. Instead, it is driven by sensory sensitivities, fear of aversive consequences, and lack of interest in food/eating. The emergence of other eating disorder symptoms  -  such as shape and weight concerns -  in the longitudinal course of ARFID is an important clinical phenomenon that is neither robustly documented nor well understood. Following ARFID history, 71% of participants in our study developed a restricting eating disorder and 29% developed a binge-spectrum eating disorder. Cognitive symptoms of other eating disorders (e.g., shape/weight concerns) onset prior to behavioral symptoms (e.g., food avoidance). Diagnostic crossover from ARFID to another eating disorder following the development of shape/weight concerns represents an interesting and important clinical phenomenon. Our findings suggest potential pathways by which ARFID may lead to the development of a subsequent eating disorder, in turn highlighting critical targets that may be intervened on to prevent this trajectory.

## Introduction

Avoidant/restrictive food intake disorder (ARFID) is a feeding/eating disorder characterized by avoidance/restriction of food intake by volume and/or variety. The core clinical feature that distinguishes ARFID from other eating disorders—specifically, anorexia nervosa and bulimia nervosa—is the absence of dietary restriction motivated by overvaluation of shape/weight. Overvaluation of shape/weight describes the undue importance of an individual’s shape/weight in influencing their self-evaluation/self-worth and is theorized to be the fundamental cognitive psychopathology underlying other eating disorders [1]. By contrast, to meet diagnostic criteria for ARFID by *DSM-5* [2], there must not be any “evidence of a disturbance in the way in which one’s body weight or shape is experienced” ([2] p. 376). Instead, dietary insufficiency in ARFID is motivated by any or all of the following: sensitivities to the sensory properties of food (e.g., smell, texture taste), fear of aversive consequences of eating (e.g., choking, vomiting), and/or lack of interest in food and/or eating [2–4]. However, researchers have described other eating disorder symptoms co-occurring with or onsetting during a course of ARFID [5–9]. Without proper attention to and assessment of symptom overlap, providers may fail to address the full eating disorder, leaving the door open for enduring nutritional and mental health consequences. For instance, ARFID treatment does not currently target shape/weight disturbance and treatment for eating disorders characterized by shape/weight disturbance does not target ARFID symptoms. Diagnostic shift presupposes that the original reasons for restriction are no longer pertinent. This assumption may not be accurate and could impede the full recovery process for either ARFID or another eating disorder.

Coupled with evidence that picky eating, a feature of ARFID, may increase risk for anorexia nervosa over time [10, 11], the emergence of additional eating disorder psychopathology – such as shape/weight concerns – among those with ARFID presents a significant clinical challenge. Becker and colleagues [5] described two cases of adolescent girls who presented with ARFID symptoms and concurrently reported or developed other eating disorder psychopathology. In one case, a 12-year-old girl characterized by the ARFID fear of aversive consequences profile (i.e., choking) lost a significant amount of weight following her choking incident. However, she ultimately developed shape/weight concerns while gaining weight during ARFID treatment and simultaneously remaining fearful of choking. Another case described a 16-year-old girl with the ARFID sensory sensitivity profile who avoided eating for extended periods due to fear of embarrassment of eating preferred foods. She later began experiencing twice-weekly binge-eating episodes, weight gain, and shape/weight concerns, and engaging in dietary restriction to lose weight, all concurrent with her selective eating [5]. In other studies, 7% of participants with ARFID reported binge eating [9], 2% reported self-induced vomiting [9], and 15% [7] to 20% [9] reported driven or excessive exercise. Findings from these studies supply preliminary evidence of shape/weight concerns onsetting concurrently or following ARFID symptoms. Thorough assessment conducted among a clinical cohort of individuals initially diagnosed with ARFID via structured clinical interviews would provide more detailed and clinically meaningful information on the prevalence of this development.

Studies of diagnostic crossover may represent one fruitful method of understanding the relations between ARFID and other eating disorders. Extant research indicates substantial diagnostic shift occurring between eating disorder diagnoses [12–15], with many well-documented configurations of crossover due to symptoms emerging (e.g., binge eating) or remitting (e.g., underweight status). Less is known about diagnostic shift from ARFID to subsequent eating disorders. Two studies documented diagnostic crossover occurring from ARFID to anorexia nervosa in 3% [6] and 12% [8, 16] of individuals. Still, others provided no evidence of shift occurring from ARFID to other eating disorders at 18-month [17], 7-year [18], and 16-year [19] follow-up. Prospective follow-up studies [6, 17], especially spanning critical age periods such as puberty (a risk factor for the development of eating disorders [20], are necessary to aid in the understanding of the course and outcomes of ARFID, including diagnostic crossover to other eating disorders.

Data characterizing how ARFID configurations change over time at the symptom-level provide critical insight into the trajectory of ARFID that syndrome-level data are unable to capture. ARFID is a serious and impairing feeding/eating disorder, onsetting earlier in life than other eating disorders [5, 21, 22] and mirroring their medical and psychological consequences [21]. Rather than waiting for the event of onset to occur – in this case, emergence of other eating disorder symptoms – high quality retrospective examination of ARFID history among those who ultimately developed other eating disorders (e.g., anorexia nervosa, bulimia nervosa) may help characterize ARFID’s course and outcomes and improve its assessment and treatment planning. To that end, the current study aimed to understand the emergence of other eating disorder symptoms and diagnostic shift among individuals with ARFID. Specifically, we sought to answer the following three questions: (a) *what* eating disorder symptoms commonly emerge in the trajectory from ARFID to a subsequent eating disorder?; (b) *in what order* do eating disorder symptoms emerge in the trajectory from ARFID to subsequent eating disorder?; and (c) *when* do eating disorder symptoms emerge in the trajectory from ARFID to subsequent eating disorder? Additionally, we examined differences in clinical characteristics between individuals presenting with restricting eating disorders and binge-spectrum eating disorders following ARFID history. Due to the paucity of available data on these questions, we did not have specific a priori hypotheses.

## Method

### Participants

We recruited participants (*N =* 35) from an undergraduate research participant pool and from the general community using flyers, postings to online social media platforms, and advertisements sent to professionals who treat eating disorders. Inclusion criteria were: (a) at least 18 years of age; (b) current or lifetime anorexia nervosa, bulimia nervosa, binge-eating disorder, or other-specified feeding or eating disorder; and (c) lifetime ARFID *preceding* the development of a subsequent eating disorder (i.e., anorexia nervosa, bulimia nervosa, binge-eating disorder, or other-specified feeding or eating disorder). In sum, eligible participants were comprised of individuals who retrospectively met criteria for ARFID and later developed another eating disorder following their course of ARFID.

### Measures

#### Nine Item ARFID Screen – Lifetime Adaptation

The Nine Item ARFID Screen (NIAS; [23]) is a self-report questionnaire assessing avoidant/restrictive eating patterns characteristic of the three ARFID profiles (i.e., sensory sensitivity [ω = 0.80], fear of aversive consequences [ω = 0.91], lack of interest in food/eating [ω = .84^1^), which each represent a subscale. To capture lifetime history of ARFID, we modified NIAS items to query about lifetime avoidant/restrictive eating (e.g., instead of “*I am a picky eater*,” we asked respondents to rate the statement “*In my lifetime, I was a picky eater*”). Each item on the NIAS is rated on a scale ranging from 0 (*Strongly disagree)* to 6 (*Strongly agree*). Subscale scores range from 0 to 15, with higher scores indicative of greater levels of avoidant/restrictive eating within that profile. We used Burton Murray and colleagues’ [26] cutoff scores of ≥ 10 for sensory sensitivity and fear of aversive consequences, and ≥ 9 for lack of interest in food/eating to screen for potential ARFID symptoms.

#### Structured Clinical Interview for DSM−5 – Research Version (SCID−5−RV) - Feeding and Eating Disorders Module

The Structured Clinical Interview for *DSM-5* – Research Version (SCID-5-RV; [27]) is a semi-structured interview used to confer *DSM-5* diagnoses. Participants completed the SCID-5-RV Feeding and Eating Disorders Module after a positive screen on the NIAS to confirm lifetime ARFID diagnosis and assess subsequent eating disorder diagnosis. Rather than assessing for current ARFID, we keyed questions to assess lifetime symptoms (e.g., rather than “*In the past month, have you been uninterested in food in general or have you kept forgetting to eat?*”, we asked participants “*Were you ever uninterested in food in general or did you ever keep forgetting to eat?*”), consistent with the SCID-5-RV’s assessment of past disorders in other diagnostic categories (e.g., past major depressive disorder). We obtained age of onset for ARFID and the subsequent eating disorder using the SCID-5-RV and queried participants about the month and year of the onset of each disorder (utilizing the techniques we describe below to mitigate memory errors). We used age, month, and year of ARFID and other eating disorder onset when setting up the Longitudinal Interval Follow-Up Evaluation – Eating Disorders Module (LIFE-EAT-3; 28).

#### The Longitudinal Interval Follow-Up Evaluation – Eating Disorders Module

The Longitudinal Interval Follow-Up Evaluation – Eating Disorders Module (LIFE-EAT-3; [28]) is a semi-structured interview that assesses the presence/absence and relative severity of diagnostic features of feeding/eating disorders over a pre-specified length of time determined by the study purpose. The pre-specified length of time for the LIFE-EAT-3 for this study varied depending on ARFID and subsequent eating disorder ages of onset derived from the SCID-5-RV. For instance, if an individual reported that their ARFID onset at age eight years in January 2000 and their anorexia nervosa onset at age 13 years in June 2005, the interview would span the inclusive 5-year period ranging from ages 8–13 years. Using the LIFE-EAT-3, we dichotomously assessed the following cognitive and behavioral symptoms of eating disorders by rating them as present or absent: body image disturbance, overvaluation of shape/weight, fear of gaining weight or becoming fat, lack of recognition of seriousness of low weight, food avoidance for reasons related to shape/weight, fasting, excessive exercise, objective binge-eating episodes, subjective binge-eating episodes, self-induced vomiting, laxative use, and diuretic use. Specific details pertaining to the administration of the LIFE-EAT-3 are outlined below (see “The Current Study”).

### Procedure

We directed interested individuals to fill out a brief, online screening survey in which they completed the NIAS and reported on their eating disorder history using a single question. We asked participants “*Has a medical professional ever diagnosed you with an eating disorder such as anorexia nervosa, bulimia nervosa, binge-eating disorder, or other-specified feeding or eating disorder?*” to distinguish individuals who had been formally diagnosed with an eating disorder from those who suspected they had an eating disorder without professional confirmation. We left “medical professional” purposefully vague to encompass whomever the participant considered appropriate, whether it be their primary care physician, a medical doctor, or a mental health professional. If individuals appeared eligible based on their responses to the online screener, we invited them to take part in an online study visit conducted via Health Insurance Portability and Accountability (HIPAA) compliant videoconferencing technology. After providing informed consent, author PEK conducted the SCID-5-RV Feeding and Eating Disorders Module to confirm eligibility, establish ARFID and subsequent eating disorder diagnosis, and ascertain ages of onset of ARFID and subsequent eating disorder diagnoses to facilitate LIFE-EAT-3 set-up. Author PEK then conducted the LIFE-EAT-3. We gave participants the option of receiving research participation credit (if applicable) or $20 for their participation. The University of Wyoming Institutional Review Board approved all study procedures.

#### Retrospective assessment of eating disorder symptoms

Considering that individuals with eating disorders often experience diagnostic shift [12–15], reliable retrospective assessment to ascertain diagnosis is critical. Fortunately, retrospective assessment of eating disorder symptoms is common [1, 29]. A major pitfall of retrospective assessment, however, concerns the extent to which respondents accurately recall and report events that occurred in the past. When individuals try to recall past events, memory errors may occur: events may be completely forgotten, events may be remembered as occurring farther back in time than they actually did (forward telescoping), and/or events may be erroneously remembered as having occurred more recently than they did [30, 31]. Fortunately, there are techniques that can be implemented to help ameliorate memory errors.

Three such techniques are bounding, the use of landmark events, and the timeline follow-back approach. Bounding is a technique that helps reduce forward telescoping errors [32]. Rather than specifying the length of time within a reference period (e.g., “*Have you experienced a binge-eating episode within the last six months?*”), the assessor provides the respondent with specific dates pertaining to that reference period (e.g., “*Have you experienced a binge-eating episode since March 15th?*”). Landmark events similarly help bound a reference period by providing respondents with salient context for that reference period [30]. Landmark events can be elicited by both the assessor (e.g., public events, such as 9/11, the summer Olympics, the Covid-19 global pandemic) and the respondent (e.g., private events, such as the start of the school year or a relationship breakup). Using landmark events to assess a reference period of interest provides a salient context for relevant symptoms and behaviors, resulting in more accurate retrieval of memories [33]. Finally, the timeline follow-back approach is commonly implemented to assess alcohol use disorders [34]. The assessor uses a calendar to help orient the respondent to the assessment period and asks the respondent to report on their daily alcohol consumption forward to the present, one day at a time. Although ratings are made on a week-by-week basis, the respondent is not queried about each week. When stability in a symptom is detected, the assessor inquiries about “change points” (e.g., “*When did that symptom change?, Did that occur before or after Christmas?, How long has this been true?*”) and makes ratings based on those change points. The timeline follow-back approach demonstrates good test-retest reliability in clinical and community samples [35]. Together, the combination of bounding, landmark events, and the timeline follow-back approach improves the accuracy of retrospective reporting over approaches that do not implement these techniques. Retrospective eating disorder assessments utilize some of the aforementioned techniques to mitigate erroneous memory reporting [1, 36, 37]. To that end, we implemented these gold-standard interviewing techniques in the current study to aid with retrospective recall.

##### The Current Study

We began by setting up a calendar with the date of the assessment inputted. The rest of the calendar populated the dates going back as long as indicated by the participant; that is, from the age of ARFID onset to the age of subsequent, other eating disorder onset. We then bounded the reference period by providing the month and year of age of onset for ARFID and the subsequent, other eating disorder diagnosis (e.g., September 2001 to October 2012). Next, we identified any public landmark events and yearly recurring events that occurred during those years and noted them on the calendar. We then inquired about any private events that would help orient the participant to the period under assessment [1]. Once the calendar was complete, we used the LIFE-EAT-3, tracing eating disorder symptom forward to the present, eliciting change points in the participant’s eating disorder trajectory. We coded each symptom dichotomously for each month, with 0 = Absence and 1 = Presence.

### Statistical analyses

#### Sample characterization and clinical characteristics for those presenting with restricting versus binge-spectrum eating disorders following ARFID history

We computed descriptive statistics to characterize the sample sex, race, age at study presentation, ARFID age of onset, ARFID NIAS profile scores , subsequent eating disorder age of onset, subsequent eating disorder diagnosis, and duration (years) between ARFID and the subsequent eating disorder diagnosis. We conducted independent samples *t-*tests to compare individuals with restricting eating disorders (*n =* 25) and binge-spectrum eating disorders (*n =* 10). The independent variable for each analysis was diagnostic group (i.e., restricting eating disorder or binge-spectrum eating disorder) and the dependent variables were ARFID age of onset, ARFID NIAS profile scores (i.e., sensory sensitivity, fear of aversive consequences, lack of interest in food/eating), subsequent eating disorder age of onset, and duration between ARFID and subsequent eating disorder.

We conducted a binary logistic regression to examine whether ARFID age of onset, the three ARFID NIAS profiles, and duration between ARFID and subsequent eating disorder were associated with a greater likelihood of a restricting eating disorder (compared to a binge-spectrum eating disorder).^2^The five predictor variables were simultaneously entered as covariates. The binary criterion variable for the logistic regression was restricting ED = 0, binge−spectrum ED = 1.

#### What eating disorder symptoms commonly emerge in the trajectory from ARFID to a subsequent eating disorder?

To elucidate which eating disorder symptoms occurred most frequently, we computed descriptive statistics to assess the presence of each eating disorder symptom on the LIFE-EAT-3, grouping symptoms into two clusters of cognitive and behavioral symptoms^3^. We present results for the overall sample and by restricting and binge-spectrum eating disorders separately.

#### In what order do eating disorder symptoms emerge in the trajectory from ARFID to a subsequent eating disorder?

We computed descriptive statistics to ascertain order (e.g., for each participant, which symptom onset first, second, third, etc.) and time (years) to each eating disorder symptom onset for the overall sample and by restricting and binge-spectrum eating disorders. For each participant, the month and year of ARFID age of onset was coded as Week 0. Each symptom was coded as present on the first week it onset. We took the average of each symptom onset (e.g., fasting onset at Week 52 for one participant, Week 104 for another, etc.) to compute time to the onset of each symptom following ARFID age of onset. This approach elucidated in what order eating disorder symptoms typically occurred (i.e., common pathways for the development of symptoms), and how long it took for each eating disorder symptom to occur following ARFID onset.

#### When Do Eating Disorder Symptoms Emerge in the Trajectory from ARFID to a Subsequent Eating Disorder?

Next, we utilized a series of paired samples *t*-tests to compare time (years) to symptom emergence between eating disorder symptoms. This approach allowed us to map the average trajectory and timeline from ARFID age of onset to subsequent eating disorder age of onset. We included censored data at the earliest possible unobserved symptom onset [39]. This approach increased statistical power because it ensured that *every* participant contributed to each comparison. Further, censoring data at the earliest possible unobserved symptom onset is a conservative approach because it assumes that although the symptom has not yet occurred, it will do so at the soonest possible opportunity (i.e., the assumption that the symptom of interest will develop immediately after the assessment period). When a participant did not experience a symptom by the last possible observation point, that symptom was coded as present just after the last possible observation point; in other words, the data point was imputed, a common method for addressing missing data in paired samples *t*-tests. Of note, we only censored data for the purpose of increasing power to detect differences in paired samples *t-*tests; all other data presented (e.g., descriptive statistics in tables) are uncensored.

## Results

### Sample characterization and clinical characteristics for those presenting with restricting versus binge-spectrum eating disorders following ARFID history

Descriptive statistics are presented in Table 1. Of the 35 participants, more than half (54%; *n =* 19) transitioned to a diagnosis of anorexia nervosa, 17% (*n =* 6) to atypical anorexia nervosa, another 17% (*n =* 6) to bulimia nervosa (the only two male participants in the sample both experienced bulimia nervosa), 9% (*n =* 3) to binge-eating disorder, and 3% (*n =* 1) to other-specified feeding/eating disorder – bulimia nervosa of low frequency. To increase statistical power to detect significant differences, these four diagnostic groups were combined to form two broader diagnostic categories – one comprised of restricting eating disorders (i.e., anorexia nervosa and atypical anorexia nervosa; *n =* 25; 71%) and the other comprised of binge-spectrum eating disorders (i.e., bulimia nervosa, binge-eating disorder, and other-specified feeding/eating disorder – bulimia nervosa of low frequency; *n =* 10; 29%). Descriptive statistics are presented for the overall sample, as well as for each subgroup.

**Table 1:** Sample characteristics and independent samples t-tests comparing restricting and binge-spectrum eating disorders

	Full Sample (*N* = 35)	Restricting Eating Disorders (*n =* 25)	Binge-spectrum Eating Disorders (*n =* 10)			
	***N*** **(%)**					
Sex (% female)	33 (94)	25 (100)	8 (80)			
Race/Ethnicity						
White	32 (91)	23 (92)	9 (90)			
Spanish Origin, or Hispanic/Latinx	1 (3)	1 (4)	0 (0)			
Middle Eastern or North African	1 (3)	1 (4)	0 (0)			
Asian	1 (3)	0 (0)	1 (10)			
Nine Item ARFID Screen Cut-Offs						
Sensory Sensitivity	27 (77)	19 (76)	8 (80)			
Fear of Aversive Consequences	21 (60)	10 (40)	6 (60)			
Lack of Interest in Food/Eating	16 (46)	16 (64)	5 (50)			
	***M*** **(SD)**			***t***	***p-*** **value**	**Cohen’s d**
Age at study presentation (years)	23.17 (5.84)	23.84 (6.50)	21.50 (3.47)	1.07	.291	.40
Age of onset ARFID (years)	8.11 (3.30)	7.76 (3.17)	9.00 (3.62)	-1.01	.322	− .38
Age of onset other eating disorder (years)	15.29 (2.97)	15.16 (2.64)	15.60 (3.81)	− .39	.698	− .15
Duration between ARFID onset and eating disorder onset (years)	7.57 (3.32)	7.52 (3.58)	7.70 (2.71)	− .14	.887	− .05
Nine Item ARFID Screen Profile Scores						
Sensory Sensitivity	11.06 (2.45)	11.04 (2.57)	11.10 (2.23)	− .07	.949	− .02
Fear of Aversive Consequences	8.66 (4.06)	8.72 (3.92)	8.50 (4.60)	.14	.887	.05
Lack of Interest in Food/Eating	9.51 (3.94)	10.32 (3.64)	7.50 (4.12)	1.99	.055	.75

*Note*. M – mean; SD – standard deviation; ARFID – avoidant/restrictive food intake disorders. Restricting eating disorders comprise anorexia nervosa and bulimia nervosa; binge-spectrum eating disorders comprise bulimia nervosa, binge-eating disorder, and other-specified feeding/eating disorder – bulimia nervosa of low frequency.

Results from independent samples *t-*tests comparing age at study presentation, ARFID age of onset, ARFID NIAS profiles (i.e., sensory sensitivity, fear of aversive consequences, lack of interest in food/eating), subsequent eating disorder age of onset, and duration between ARFID and subsequent eating disorder between restricting and binge-spectrum eating disorder subgroups are in Table 1. There were no significant differences between individuals with restricting eating disorders and binge-spectrum eating disorders on any of these descriptive variables.

A binary logistic regression examined whether age of ARFID onset, duration between ARFID and subsequent eating disorder, and each of the three ARFID profiles (i.e., sensory sensitivity, fear of aversive consequences, lack of interest in food/eating) were uniquely associated with likelihood of a restricting eating disorder versus binge-spectrum eating disorder. Results indicated that severity in the ARFID lack of interest profile was associated with almost one and a half times the likelihood of a restricting eating disorder than binge-spectrum eating disorder (*B* [*SE*] = 0.31 [0.14], Wald χ2 = 5.16, OR = 1.34 [1.04–1.77], *p =* .023). Age of ARFID onset (*B* [*SE*] = 0.31 [0.16], Wald χ2 = 3.58, OR = 1.34 [0.99–1.87], *p =* .058), duration between ARFID and subsequent eating disorder (*B* [*SE*] = 0.21 [0.16], Wald χ2 = 1.61, OR = 1.23 [0.89–1.69], *p =* .205), the ARFID sensory sensitivity profile (*B* [*SE*] = 0.18 [0.21], Wald χ2 = 0.74, OR = 1.20 [0.79–1.82], *p =* .390), and the ARFID fear of aversive consequences profile (*B* [*SE*] = 0.03 [0.12], Wald χ2 = 0.09, OR = 1.03 [0.83–1.23], *p =* .771) were not uniquely associated with likelihood of a restricting eating disorder compared to a binge-spectrum eating disorder.

### What eating disorder symptoms commonly emerge in the trajectory from ARFID to a subsequent eating disorder?

Table 2 describes each LIFE-EAT-3 eating disorder symptom following ARFID onset. Regarding cognitive symptoms, almost all participants (97%) experienced overvaluation of shape/weight. The next most common cognitive symptom was body image disturbance (83%), followed by fear of gaining weight or becoming fat (80%). Only four participants (11%; all of whom had anorexia nervosa) experienced lack of recognition of seriousness of low body weight. With respect to behavioral symptoms, food avoidance was the most common (occurring in 63% of participants), followed by fasting (49%), driven/excessive exercise (37%), and objective binge eating (31%). Fewer participants reported subjective binge eating (20%) or purging (14%), which included self-induced vomiting, laxative use, and diuretic use.

**Table 2:** Presence of eating disorder symptoms and mean/standard deviation years to onset following ARFID

	Full Sample (*N =* 35)		Restricting Eating disorders (*n =* 25)		Binge-Spectrum Eating Disorders (*n =* 10)	
**Cognitive Symptoms**	*N* (%)	*M* (*SD*)	*N* (%)	M (*SD*)	*N* (%)	M (*SD*)
Body Image Disturbance	29 (83)	4.62 (2.97)	21 (84)	4.82 (3.18)	8 (80)	4.10 (2.45)
Overvaluation	34 (97)	4.81 (2.51)	24 (96)	4.82 (2.79)	10 (100)	4.77 (1.81)
Fear of Gaining Weight/Becoming Fat	28 (80)	4.85 (2.77)	21 (84)	4.49 (3.02)	7 (70)	5.93 (1.45)
Lack of Recognition of Seriousness of Low Body Weight	4 (11)	9.05 (3.99)	4 (16)	9.04 (3.99)	0 (0)	N/A
**Behavioral Symptoms**	*N* (%)	*M* (*SD*)	*N* (%)	M (*SD*)	*N* (%)	M (*SD*)
Food Avoidance	22 (63)	5.78 (3.32)	19 (76)	5.71 (5.93)	3 (30)	6.19 (2.60)
Objective Binge Episodes	11 (31)	7.34 (4.52)	4 (16)	8.94 (5.93)	7 (70)	6.42 (3.71)
Subjective Binge Episodes	7 (20)	7.64 (3.14)	4 (16)	5.85 (2.34)	3 (30)	10.01 (2.54)
Fasting	17 (49)	6.43 (2.74)	14 (560	6.33 (2.67)	3 (30)	6.93 (3.60)
Excessive Exercise	13 (37)	6.40 (3.68)	11 (44)	5.95 (3.75)	2 (20)	8.88 (2.60)
Self-Induced Vomiting	4 (11)	5.14 (4.28)	4 (16)	5.14 (4.28)	0 (0)	N/A
Laxative Use	2 (6)	6.50 (5.45)	2 (8)	6.50 (5.45)	0 (0)	N/A
Diuretic Use	1 (3)	8.67 (N/A)	1 (4)	8.67 (N/A)	0 (0)	N/A

*Note*. M – mean; SD – standard deviation; N/A – not applicable

**Fig. 1:**
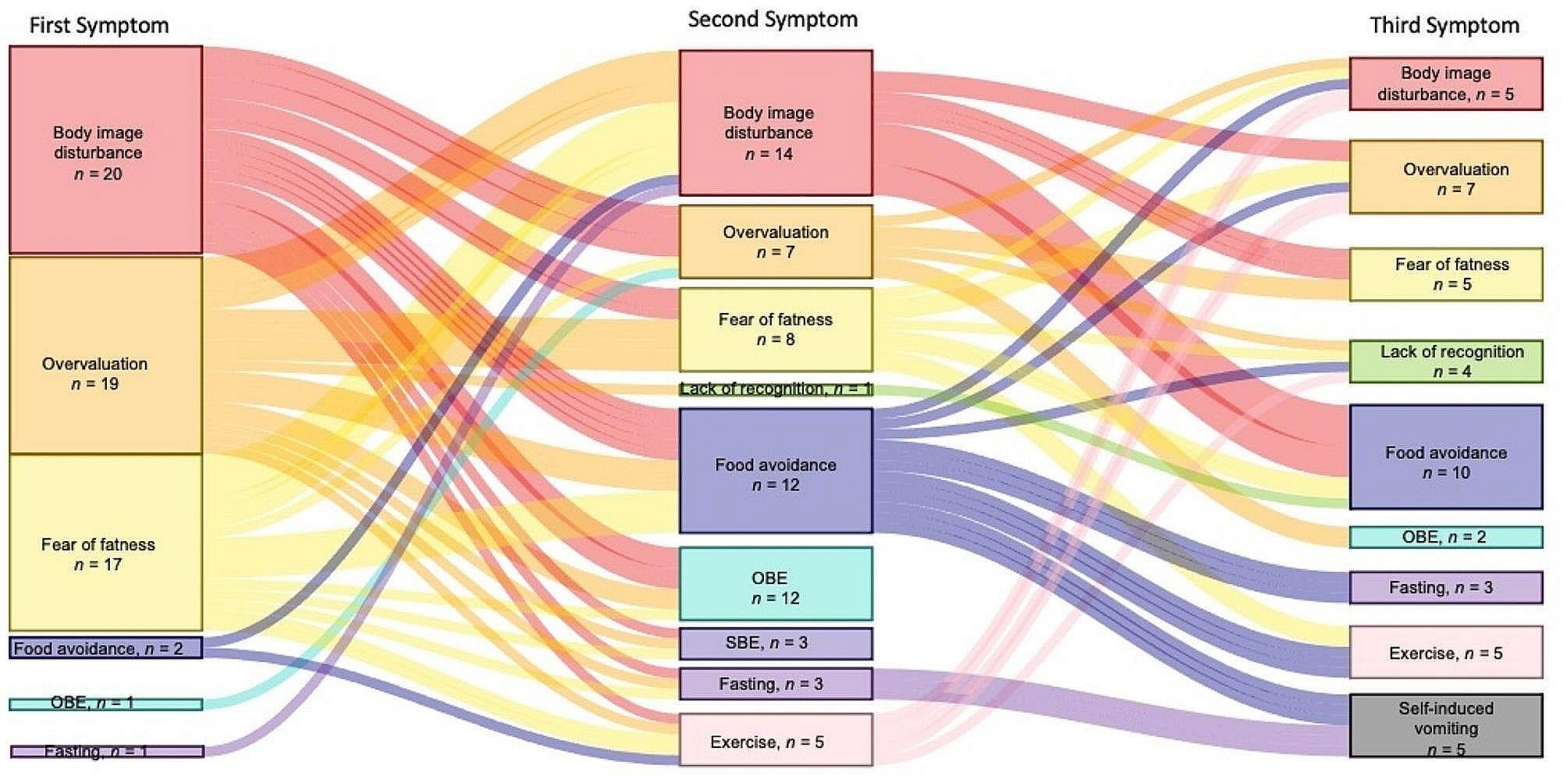
First, second and third symptoms to develop following ARFID age of onset. *Note*. OBE – objective binge eating; SBE – subjective binge eating. A Sankey diagram includes nodes and arcs. As transitions occur, each arc flows from its source node to its target note. The size of each node and width of each arc represent the number of participants, thus indicating the magnitude of flow. For instance, a node with five participants experiencing a trajectory would be half as tall as a node with ten participants. The numbers depicted on the Sankey diagram indicate the number of individuals who experienced any given symptom first, second or third

### In what order do eating disorder symptoms emerge in the trajectory from ARFID to a subsequent eating disorder?

Figure 1 depicts a Sankey diagram outlining the development of the first, second, and third cognitive and behavioral symptoms to onset following ARFID. Most participants (*n =* 30; 86%) experienced a cognitive symptom first, though a small subset (11%, *n =* 4) experienced a behavioral symptom first, and just one participant (3%) endorsed a cognitive (i.e., fear of gaining weight or becoming fat) and behavioral (i.e., food avoidance) symptom occurring first concurrently. Of the cognitive symptoms, overvaluation of shape/weight was most often the first to emerge (46% of participants), followed by fear of gaining weight or becoming fat (43%), and body image disturbance (34%). Of the behavioral symptoms, food avoidance emerged first for 6% (*n =* 2), followed by objective binge eating in 3% (*n =* 1), and fasting in another 3% (*n =* 1). Lack of recognition of seriousness of low body weight, subjective binge episodes, driven/excessive exercise, and purging (including self-induced vomiting, laxative use, diuretic use) did not onset first for any participants.

### When do eating disorder symptoms emerge in the trajectory from ARFID to a subsequent eating disorder?

**Fig. 2:**
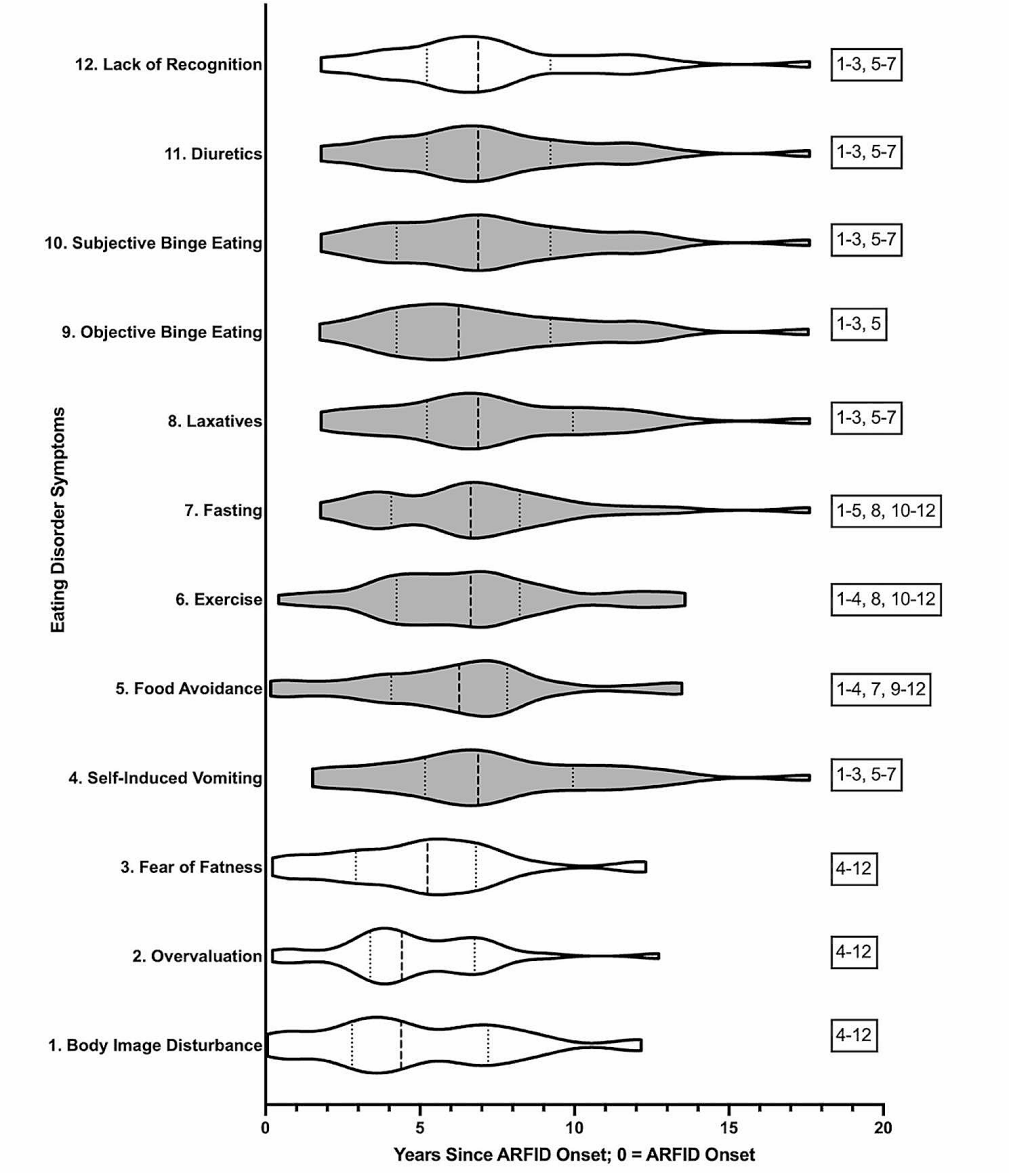
Years to emergence of eating disorders following ARFID data including censored data at the earliest possible unobserved symptom onset. *Note*. Symptoms shaded in gray are behavioral symptoms. Unshaded symptoms are cognitive symptoms. From bottom to top, symptoms are presented in the average order at which they onset. The numbers to the right of the violin plots indicate symptoms that were significantly different from one another at *p* < .05. For instance, body image disturbance differed significantly from all symptoms except overvaluation and fear of fatness. Dotted lines represent the quartiles and dashed lines represent the median

Table 2 describes the average number of years to symptom onset for the overall sample and each diagnostic subgroup. Table 3 provides effect sizes and indicates statistical significance for each of the paired-samples *t-*tests comparing years to symptom emergency for each reported symptom. Figure 2 depicts violin plot illustrating the distribution of each symptom to emerge following ARFID onset.

**Table 3:** Effect sizes for paired samples t-tests comparing years to each symptom onset

	Body Image Disturbance	Overvaluation	Fear of Gaining Weight/Becoming Fat	Lack of Recognition of Seriousness of Low Body Weight	Food Avoidance	Objective Binge Episodes	Subjective Binge Episodes	Fasting^1^	Excessive Exercise	Self-Induced Vomiting	Laxative Use	Diuretic Use
Body Image Disturbance	-											
Overvaluation	0.07	-										
Fear of Gaining Weight/Becoming Fat	-0.09	-0.18	-									
Lack of Recognition of Seriousness of Low Body Weight	**-0.95**	**-1.00**	**-0.87**	-								
Food Avoidance	**-0.60**	**-0.58**	**-0.54**	**0.68**	-							
Objective Binge Episodes	**-0.80**	**-0.85**	**-0.70**	0.11	**-0.47**	-						
Subjective Binge Episodes	**-0.87**	**-0.91**	**-0.80**	-0.02	**-0.64**	-0.19	-					
Fasting	**-0.84**	**-0.87**	**-0.76**	**0.40**	**-0.45**	0.25	**0.42**	-				
Excessive Exercise	**-0.65**	**-0.73**	**-0.66**	**0.48**	-0.33	0.26	**0.44**	0.05	-			
Self-Induced Vomiting	**-0.91**	**-0.95**	**-0.83**	-0.11	**-0.73**	-0.25	-0.12	**-0.47**	**-0.47**	-		
Laxative Use	**-0.96**	**-1.00**	**-0.86**	-0.18	**-0.75**	-0.24	-0.11	**-0.50**	**-0.53**	0.02	-	
Diuretic Use	**-0.99**	**-1.04**	**-0.90**	-0.29	**-0.73**	-0.20	-0.08	**-0.50**	**-0.54**	0.02	0.02	-

*Note*. Table demonstrate effect sizes when including censored data at the earliest possible unobserved symptom onset. Values represent Cohen’s *d* for paired samples *t-*tests. Effect sizes are bolded to demonstrate statistical significance at *p* < .05. Effect sizes should be interpreted from right (column) to left (row). For instance, the first column indicates that body image followed overvaluation (due to a positive effect size), whereas it preceded all other symptoms (indicated by a negative effect size). Negative effect size signs indicate symptoms that preceded the reference symptom; positive effect size signs indicate symptoms that followed the reference symptom

No significant differences emerged between the average years to symptom onset for three of the four cognitive symptoms (i.e., body image disturbance, overvaluation of shape/weight, fear of gaining weight or becoming fat). Onset of all three of these cognitive symptoms preceded onset of all behavioral symptoms (i.e., food avoidance, objective binge-eating episodes, subjective binge-eating episodes, fasting, driven/excessive exercise, self-induced vomiting, laxative use, and diuretic use). The only exception was lack of recognition of seriousness of low body weight, a cognitive symptom that occurred significantly later than body image disturbance, overvaluation of shape/weight, and fear of gaining weight or becoming fat. Lack of recognition of seriousness of low body weight also followed behavioral symptoms of food avoidance, fasting, and driven/excessive exercise; the only of the cognitive symptoms to occur following onset of behavioral symptoms.

With respect to the behavioral symptoms, food avoidance preceded the onset of all other behavioral symptoms except for driven/excessive exercise. There were similarly no significant differences between time to objective or subjective binge-eating episodes and other behavioral symptoms, with the exception that subjective binge-eating episodes onset following fasting and driven/excessive exercise. Fasting and exercise, though themselves not significantly different in time to onset, preceded all three forms of purging (self-induced vomiting, laxative use, diuretic use). The three forms of purging did not significantly differ from one another in years to onset.

## Discussion

We sought to understand the emergence of eating disorder symptoms and diagnostic shift among individuals with ARFID through a retrospective examination of their illness course and outcome. The diagnostic stability of other eating disorders is known to fluctuate over time. Understanding these symptom fluctuations is critical for understanding how eating disorders evolve. Most individuals with ARFID (71%) transitioned to restricting eating disorders (i.e., anorexia nervosa and atypical anorexia nervosa), while the remainder (29%) developed binge-spectrum eating disorders (i.e., bulimia nervosa and binge-eating disorder). The most common symptoms to develop in the transition from ARFID to subsequent eating disorders were cognitive symptoms of body image disturbance, overvaluation of shape/weight, and fear of gaining weight or becoming fat. The most frequent behavioral symptoms were food avoidance related to shape/weight and fasting. Cognitive symptoms onset first – on average, slightly less than five years following the onset of ARFID – and were followed by behavioral symptoms. The first behavioral symptom to emerge was food avoidance related to shape/weight (approximately six years following ARFID onset). To our knowledge, no prior studies have characterized this trajectory. Though prospective replication of these findings utilizing larger samples is critically needed, these findings highlight the protracted trajectory of eating disorder symptom emergence following ARFID onset.

### Syndrome-level trajectory from ARFID to subsequent eating disorders

Shift from ARFID to restricting eating disorders was more common than shift to binge-spectrum eating disorders. This may be attributable to the fact that ARFID and anorexia nervosa share the hallmark feature of dietary restriction. Most studies of diagnostic crossover in eating disorders [12, 13, 15, 40] suggest that the likelihood of transitioning from a restricting eating disorder to a binge-spectrum eating disorder is almost twice the likelihood of the reverse ($\sim$50% [41] and $\sim$27% [15], respectively). Studies to date have only documented crossover from ARFID to anorexia nervosa [6, 8, 42]. Because little is known about diagnostic crossover from ARFID to subsequent eating disorders, it is unclear whether individuals with ARFID who are subsequently diagnosed with anorexia nervosa will follow the trajectory of crossover commonly delineated in the eating disorders literature – that is, whether they will subsequently develop a binge-spectrum eating disorder – or if the presence of ARFID preceding anorexia nervosa alters that path in any meaningful way.

One possibility is that the presence of low homeostatic and hedonic hunger among individuals with ARFID – particularly characterized by the lack of interest profile [3, 43]– may serve as a protective factor for the development of binge eating, which is often preceded by hunger [44]. Further, the ARFID lack of interest in food/eating profile is associated with low levels of anticipatory pleasure [45]. In the current study, the ARFID lack of interest profile uniquely predicted greater likelihood of developing a restricting eating disorder (versus a binge-spectrum eating disorder). Coupled with the large effect demonstrated in the difference between restricting and binge-spectrum eating disorders on scores on this profile (Table 1), this finding may suggest that ARFID lack of interest could be a noteworthy precursor to a restricting eating disorder, and may protect against later development of a binge-spectrum eating disorder. By contrast, long-standing patterns of irregular eating and fasting through both ARFID and later transition to anorexia nervosa may contribute to increased risk for binge eating [5, 46]. Future research should conduct longer prospective examinations to further characterize this trajectory.

### Symptom-level trajectory from ARFID to subsequent eating disorders

#### Cognitive symptoms

A vast majority of participants experienced a cognitive – rather than a behavioral – eating disorder symptom as their first subsequent eating disorder symptom to onset following ARFID. This finding suggests that one pathway by which ARFID could lead to another eating disorder is by the emergence of cognitive, followed by behavioral symptoms. Cognitive symptoms of eating disorders reflect the underlying thought processes and attitudes that later contribute to the development of behaviors [47–50]. Overvaluation of shape/weight is the core cognitive symptom that distinguishes ARFID from anorexia nervosa and bulimia nervosa. In fact, ARFID cannot occur in the context of “a disturbance in the way in which one’s body weight or shape is experienced” (2; p. 376), defined as a distorted perception of one’s shape/weight that often manifest in behavioral symptoms [6]. Almost all participants (97%) experienced overvaluation of shape/weight in their trajectory from ARFID to a subsequent eating disorder, which is consistent with the nature of the sample (i.e., individuals with eating disorder) and the transdiagnostic model of eating disorders, which centers overvaluation of shape/weight as the core psychopathology of eating disorders [51]. Half of these participants reported overvaluation of shape/weight as the first symptom to onset following ARFID. The one participant who did not experience onset of this symptom developed anorexia nervosa, where overvaluation of shape/weight is not always present.

Body image disturbance, overvaluation of shape/weight, and fear of gaining weight or becoming fat (together henceforth referred to as “shape/weight concerns”) did not significantly differ from one another in their time to emergence. Lack of recognition of seriousness of low body weight, although a cognitive symptom, occurred following shape/weight concerns. Behavioral symptoms onset an average of one year and eight months following cognitive symptoms. With four exceptions, behaviors occurred in response to existing cognitions, engendering the possibility that behaviors are caused by cognitions (i.e., predisposition/vulnerability model; [52]). Another possibility is that cognitions and behaviors are pathoplastic – that cognitions influence the manifestation (e.g., expression, severity, course) of behaviors, rather than having a causal etiological role (i.e., pathoplasty model; [53]). One potential implication of the predisposition/vulnerability model is that it may be important to intervene on and address cognitive symptoms at their earliest onset to prevent the development of subsequent eating disorder behaviors. In contrast, evidence for the pathoplasty model would highlight the utility of tailoring early interventions to target both cognitions *and* behaviors. Still another explanation is that the relation between cognitions and behaviors in the context of shift from ARFID to other eating disorders is far more complex than either the vulnerability or pathoplasty model suggest, and that the two symptoms may interact in a bidirectional or reciprocal manner. For instance, changes in endocrine (e.g., higher levels of CCK; [54]) and brain function over the course of ARFID may interact with environmental factors (e.g., societal influences on shape/weight, peer pressure) to influence later symptom development. Overall, future research is needed to replicate these findings and further clarify the relation between cognitive and behavioral symptoms in the development of eating disorders following ARFID.

#### Behavioral symptoms

Food avoidance was the first behavioral symptom to onset, preceding *all* other behavioral symptoms except driven/excessive exercise. It could be argued that if ARFID is predominantly characterized by one eating behavior, that behavior is food avoidance. In the trajectory from ARFID to a subsequent eating disorder, it is the function, rather than the presence, of food avoidance that changes, perhaps providing a plausible explanation for why this is the first behavioral symptom to emerge. In other words, rather than being motivated by sensory sensitivities, fear of aversive consequences, or lack of interest in food/eating, food avoidance shifts to being driven by shape/weight concerns, rather than exhibiting a *de novo* onset. It is even possible that food avoidance becomes driven by a combination of these underlying motivators (i.e., sensory sensitivities *and* shape/weight concerns), which has been documented in case series of patients with ARFID [5]. For instance, a patient with sensory sensitivity to the texture of food may begin also restricting their food intake for reasons related to shape/weight. Moreover, patients with ARFID who begin avoiding/restricting their food intake due to sensory sensitivities may later develop lack of interest in food/eating as they become tired of consistently eating the same foods. This finding provides evidence of continuity across eating disorder diagnoses.

Regarding other behavioral symptoms, although objective binge eating did not differ in its time to onset from fasting and driven/excessive exercise, subjective binge eating followed the onset of these symptoms. Fasting and driven/excessive exercise, though themselves not significantly different, preceded purging (i.e., self-induced vomiting, laxative use, diuretic use), and individual purging symptoms did not differ in their time to onset. Of note, very few participants reported purging (14%). The ARFID fear of aversive consequences profile (which was present for almost half the sample), insofar as it includes fear of vomiting following food intake, may reasonably serve to protect against the most common form of purging – self-induced vomiting [55] – in the trajectory to a subsequent eating disorder. Future research should ascertain whether crossover from ARFID to restricting eating disorders occurs more frequently than crossover to binge-spectrum eating disorders (i.e., whether the current findings are generalizable to the population of ARFID who transition to other eating disorders).

### Limitations

Study findings should be interpreted with limitations in mind. The sample was homogenous with respect to age, sex, and race/ethnicity; therefore, it is unclear how findings generalize to other groups, particularly males and underrepresented individuals. Epidemiological studies indicate that eating disorders are more common among females than males [56, 57], except in ARFID, which has a more equal sex distribution [7, 8]. Future research should focus on minoritized individuals with ARFID given the dearth of research in this area, despite unique risk factors at play for these populations [58, 59]. Our sample size was small and limitations associated with an underpowered study can have implications that affect the validity and generalizability of results. Finally, there may be limited external validity and generalizability, as the small sample may not have adequately represented the population of interest or may have limited variability in the constructs under study. Therefore, caution should be exercised when generalizing findings. Further, we did not assess ARFID symptoms following the onset of the subsequent eating disorder, so these findings cannot speak to the overlap between ARFID symptoms and subsequent eating disorder symptoms. ARFID, subsequent eating disorder diagnosis, and symptom emergence were assessed retrospectively, which may limit the accuracy and reliability of results. Future studies using prospective data collection can provide a more detailed assessment of the course and outcome of ARFID and are necessary to follow participants over time and collect relevant data at regular intervals.

## Conclusions

Limitations notwithstanding, this is the longest retrospective assessment period (spanning 2–17 years) to document crossover from ARFID to other eating disorders using methods geared toward improving the accuracy of retrospectively reported eating disorder symptoms (e.g., bounding, landmark events, timeline follow-back approach). Diagnostic crossover from ARFID to another eating disorder following the development of shape/weight concerns represents an interesting and important clinical phenomenon. Our findings suggest potential pathways by which ARFID may lead to the development of a subsequent eating disorder, in turn highlighting critical targets that may be intervened on to prevent this trajectory.

## Data Availability

The data that support the findings of this study are available from the corresponding, PEK, upon reasonable request.

## Footnotes

1 We report McDonald’s omega, as opposed to Cronbach’s alpha, as an indicator of unidimensional scale reliability due to assumptions underlying the calculation of alpha that are unlikely to be met [24]. A McDonald’s omega value > 0.75 is preferred [25].

2 To confirm that there was no problematic multicollinearity among the predictor variables, their interrelations were examined using Pearson zero-order correlations. All correlations fell below the suggested 0.90 threshold indicative of problematic multicollinearity (Pearson *r*’s ranged from 0.03 to 0.57; [38]).

3 Cognitive symptoms included body image disturbance, overvaluation of shape/weight, fear of gaining weight or becoming fat, and lack of recognition of seriousness of low weight. Behavioral symptoms included food avoidance, fasting, excessive exercise, objective binge-eating episodes, subjective binge-eating episodes, self-induced vomiting, laxative use, and diuretic use.

## References

[1] Fairburn CG, Cooper Z, Shafran R, Wilson GT (2008). Eating disorders: a transdiagnostic protocol. Clinical handbook of psychological disorders: a step-by-step treatment manual.

[2] American Psychiatric Association, editor (2022). Diagnostic and statistical manual of mental disorders: DSM-5-TR. Fifth edition, text revision.

[3] Thomas JJ, Lawson EA, Micali N, Misra M, Deckersbach T, Eddy KT (2017). Avoidant/Restrictive food intake disorder: a three-Dimensional Model of Neurobiology with implications for etiology and treatment. Curr Psychiatry Rep.

[4] Thomas JJ, Eddy K (2019). Cognitive-behavioral therapy for avoidant/restrictive food intake disorder: children, adolescents, and adults.

[5] Becker KR, Breithaupt L, Lawson EA, Eddy KT, Thomas JJ (2020). Co-occurrence of Avoidant/Restrictive Food Intake Disorder and traditional eating psychopathology. J Am Acad Child Adolesc Psychiatry.

[6] Kambanis PE, Tabri N, McPherson I, Gydus J, Kuhnle MC, Stern CM et al. Prospective two-year course and predictors of outcome in avoidant/restrictive food intake disorder. Under review. 10.1016/j.jaac.2024.04.010 PMC13006878 38718975

[7] Nicely TA, Lane-Loney S, Masciulli E, Hollenbeak CS, Ornstein RM (2014). Prevalence and characteristics of avoidant/restrictive food intake disorder in a cohort of young patients in day treatment for eating disorders. J Eat Disord.

[8] Norris ML, Robinson A, Obeid N, Harrison M, Spettigue W, Henderson K (2014). Exploring avoidant/restrictive food intake disorder in eating disordered patients: a descriptive study. Intl J Eat Disorders.

[9] Strandjord SE, Sieke EH, Richmond M, Rome ES (2015). Avoidant/Restrictive food intake disorder: illness and hospital course in patients hospitalized for nutritional insufficiency. J Adolesc Health.

[10] Herle M, Stavola BD, Hübel C, Abdulkadir M, Ferreira DS, Loos RJF (2020). A longitudinal study of eating behaviours in childhood and later eating disorder behaviours and diagnoses. Br J Psychiatry.

[11] Jacobi C, Schmitz G, Agras WS (2008). Is picky eating an eating disorder?. Int J Eat Disord.

[12] Castellini G, Lo Sauro C, Mannucci E, Ravaldi C, Rotella CM, Faravelli C (2011). Diagnostic crossover and outcome predictors in eating disorders according to DSM-IV and DSM-V proposed criteria: a 6-year follow-up study. Psychosom Med.

[13] Eddy KT, Dorer DJ, Franko DL, Tahilani K, Thompson-Brenner H, Herzog DB (2008). Diagnostic crossover in anorexia nervosa and bulimia nervosa: implications for DSM-V. Am J Psychiatry.

[14] Eddy KT, Swanson SA, Crosby RD, Franko DL, Engel S, Herzog DB (2010). How should DSM-V classify eating disorder not otherwise specified (EDNOS) presentations in women with lifetime anorexia or bulimia nervosa?. Psychol Med.

[15] Tozzi F, Thornton LM, Klump KL, Fichter MM, Halmi KA, Kaplan AS (2005). Symptom fluctuation in eating disorders: correlates of diagnostic crossover. Am J Psychiatry.

[16] Norris ML, Santos A, Obeid N, Hammond NG, Valois DD, Isserlin L (2020). Characteristics and clinical trajectories of patients meeting criteria for avoidant/restrictive food intake disorder that are subsequently reclassified as anorexia nervosa. Eur Eat Disorders Rev.

[17] Breithaupt L, Kahn DL, Slattery M, Plessow F, Mancuso C, Izquierdo A (2022). Eighteen-month Course and Outcome of adolescent restrictive eating disorders: persistence, crossover, and recovery. J Clin Child Adolesc Psychol.

[18] Nakai Y, Nin K, Noma S, Hamagaki S, Takagi R, Teramukai S (2017). Clinical presentation and outcome of avoidant/restrictive food intake disorder in a Japanese sample. Eat Behav.

[19] Lange CRA, Ekedahl Fjertorp H, Holmer R, Wijk E, Wallin U (2019). Long-term follow-up study of low-weight avoidant restrictive food intake disorder compared with childhood-onset anorexia nervosa: Psychiatric and occupational outcome in 56 patients. Int J Eat Disord.

[20] Klump KL (2013). Puberty as a critical risk period for eating disorders: a review of human and animal studies. Horm Behav.

[21] Aulinas A, Marengi DA, Galbiati F, Asanza E, Slattery M, Mancuso CJ (2020). Medical comorbidities and endocrine dysfunction in low-weight females with avoidant/restrictive food intake disorder compared to anorexia nervosa and healthy controls. Int J Eat Disord.

[22] Zanna V, Criscuolo M, Mereu A, Cinelli G, Marchetto C, Pasqualetti P (2021). Restrictive eating disorders in children and adolescents: a comparison between clinical and psychopathological profiles. Eat Weight Disord.

[23] Zickgraf HF, Ellis JM (2018). Initial validation of the Nine Item Avoidant/Restrictive Food Intake disorder screen (NIAS): a measure of three restrictive eating patterns. Appetite.

[24] Hayes AF, Coutts JJ (2020). Use Omega rather than Cronbach’s alpha for estimating reliability. But… Communication Methods Measures.

[25] Reise SP (2012). Invited paper: the rediscovery of Bifactor Measurement models. Multivar Behav Res.

[26] Burton Murray H, Dreier MJ, Zickgraf HF, Becker KR, Breithaupt L, Eddy KT (2021). Validation of the nine item ARFID screen (NIAS) subscales for distinguishing ARFID presentations and screening for ARFID. Int J Eat Disord.

[27] First MB, Williams JBW, Karg RS, Spitzer RL. User’s guide for the SCID-5-CV Structured Clinical Interview for DSM-5® disorders: Clinical version. Arlington, VA, US: American Psychiatric Publishing, Inc.; 2016. xii, 158 p. (User’s guide for the SCID-5-CV Structured Clinical Interview for DSM-5® disorders: Clinical version).

[28] Eddy KT, Murray HB, Thomas JJ. Longitudinal Interval Follow-Up Evaluation Eating and Feeding Disorders Version (LIFE-EAT-3). Adapted from Keller 1987. 2015.

[29] Fairburn CG, Beglin SJ (1994). Assessment of eating disorders: interview or self-report questionnaire?. Int J Eat Disord.

[30] Loftus EF, Marburger W (1983). Since the eruption of Mt. St. Helens, has anyone beaten you up? Improving the accuracy of retrospective reports with landmark events. Mem Cognit.

[31] Sudman S, Bradburn NM (1973). Effects of Time and Memory factors on response in surveys. J Am Stat Assoc.

[32] Brennan M, Chan J, Hini D, Esslemont D. Improving the Accuracy of Recall Data: A Test of Two Procedures. 1996.

[33] Friedenreich CM (1994). Improving long-term recall in epidemiologic studies. Epidemiology.

[34] Sobell LC, Maisto SA, Sobell MB, Cooper AM (1979). Reliability of alcohol abusers’ self-reports of drinking behavior. Behav Res Ther.

[35] Sobell LC, Sobell MB, Leo GI, Cancilla A (1988). Reliability of a timeline method: assessing normal drinkers’ reports of recent drinking and a comparative evaluation across several populations. Br J Addict.

[36] De Young KP, Anderson DA (2017). An interactive, graphical Tool for retrospectively assessing Symptom frequency and severity: an illustration with eating disorder behaviors, Body Weight, and stress. Assessment.

[37] Eddy KT, Tabri N, Thomas JJ, Murray HB, Keshaviah A, Hastings E (2017). Recovery from Anorexia Nervosa and Bulimia Nervosa at 22-Year Follow-Up. J Clin Psychiatry.

[38] Tabachnick BG, Fidell LS (2013). Using multivariate statistics.

[39] Collett D (2015). Modelling Survival Data in Medical Research.

[40] Fichter MM, Quadflieg N, Crosby RD, Koch S (2017). Long-term outcome of anorexia nervosa: results from a large clinical longitudinal study. Int J Eat Disord.

[41] Strober M, Freeman R, Morrell W (1997). The long-term course of severe anorexia nervosa in adolescents: survival analysis of recovery, relapse, and outcome predictors over 10–15 years in a prospective study. Int J Eat Disord.

[42] Norris ML, Spettigue W, Hammond NG, Katzman DK, Zucker N, Yelle K (2018). Building evidence for the use of descriptive subtypes in youth with avoidant restrictive food intake disorder. Int J Eat Disord.

[43] Brigham KS, Manzo LD, Eddy KT, Thomas JJ (2018). Evaluation and Treatment of Avoidant/Restrictive Food Intake Disorder (ARFID) in adolescents. Curr Pediatr Rep.

[44] Pinaquy S, Chabrol H, Simon C, Louvet JP, Barbe P (2003). Emotional eating, alexithymia, and binge-eating disorder in obese women. Obes Res.

[45] Dolan SC, Kambanis PE, Stern CM, Becker KR, Breithaupt L, Gydus J (2023). Anticipatory and consummatory pleasure in avoidant/restrictive food intake disorder. J Eat Disord.

[46] Haedt-Matt AA, Keel PK (2011). Revisiting the affect regulation model of binge eating: a meta-analysis of studies using ecological momentary assessment. Psychol Bull.

[47] Dingemans AE, Spinhoven P, van Furth EF (2006). Maladaptive core beliefs and eating disorder symptoms. Eat Behav.

[48] Gongora VC, Derksen JJL, van Der Staak CPF (2004). The role of core beliefs in the specific cognitions of bulimic patients. J Nerv Ment Dis.

[49] Hughes EK, Kerr JA, Patton GC, Sawyer SM, Wake M, Le Grange D (2019). Eating disorder symptoms across the weight spectrum in Australian adolescents. Int J Eat Disord.

[50] Legenbauer T, Schütt-Strömel S, Hiller W, Vocks S (2011). Predictors of improved eating behaviour following body image therapy: a pilot study. Eur Eat Disord Rev.

[51] Fairburn CG (2008). Cognitive behavior therapy and eating disorders.

[52] Clark LA (2005). Temperament as a unifying basis for personality and psychopathology. J Abnorm Psychol.

[53] Bauer BW, Capron D. Pathoplasty Model. In: Zeigler-Hill V, Shackelford TK, editors. Encyclopedia of Personality and Individual Differences [Internet]. Cham: Springer International Publishing; 2017 [cited 2024 Jan 11]. pp. 1–2. 10.1007/978-3-319-28099-8_921-1.

[54] Burton Murray H, Becker KR, Harshman S, Breithaupt L, Kuhnle M, Dreier MJ (2022). Elevated fasting satiety-promoting cholecystokinin (CCK) in Avoidant/Restrictive food intake disorder compared to healthy controls. J Clin Psychiatry.

[55] Forney KJ, Buchman-Schmitt JM, Keel PK, Frank GKW (2016). The medical complications associated with purging. Int J Eat Disord.

[56] Hoek HW (2006). Incidence, prevalence and mortality of anorexia nervosa and other eating disorders. Curr Opin Psychiatry.

[57] Striegel-Moore RH, Bulik CM (2007). Risk factors for eating disorders. Am Psychol.

[58] Egbert AH, Hunt RA, Williams KL, Burke NL, Mathis KJ (2022). Reporting racial and ethnic diversity in eating disorder research over the past 20 years. Int J Eat Disord.

[59] Goel NJ, Jennings Mathis K, Egbert AH, Petterway F, Breithaupt L, Eddy KT (2022). Accountability in promoting representation of historically marginalized racial and ethnic populations in the eating disorders field: a call to action. Int J Eat Disord.

